# A combined cross-sectional analysis and case-control study evaluating tick-borne encephalitis vaccination coverage, disease and vaccine effectiveness in children and adolescents, Switzerland, 2005 to 2022

**DOI:** 10.2807/1560-7917.ES.2024.29.18.2300558

**Published:** 2024-05-02

**Authors:** Kyra D Zens, Ekkehardt Altpeter, Monica N Wymann, Annora Mack, Nora B Baer, Sarah R Haile, Robert Steffen, Jan S Fehr, Phung Lang

**Affiliations:** 1 Epidemiology, Biostatistics and Prevention Institute, Department of Public and Global Health, University of Zurich, Zurich, Switzerland; 2 Institute for Experimental Immunology, University of Zurich, Zurich, Switzerland; 3 Communicable Diseases Division, Swiss Federal Office of Public Health (FOPH), Bern, Switzerland; 4 Epidemiology, Biostatistics and Prevention Institute, Department of Epidemiology, University of Zurich, Zurich, Switzerland

**Keywords:** Tick-borne encephalitis, Tick-borne disease, Vaccine effectiveness, Infectious disease, Epidemiology, Paediatric vaccination, Immunity, Switzerland

## Abstract

**Background:**

Tick-borne encephalitis (TBE) is a severe, vaccine-preventable viral infection of the central nervous system. Symptoms are generally milder in children and adolescents than in adults, though severe disease does occur. A better understanding of the disease burden and duration of vaccine-mediated protection is important for vaccination recommendations.

**Aim:**

To estimate TBE vaccination coverage, disease severity and vaccine effectiveness (VE) among individuals aged 0–17 years in Switzerland.

**Methods:**

Vaccination coverage between 2005 and 2022 was estimated using the Swiss National Vaccination Coverage Survey (SNVCS), a nationwide, repeated cross-sectional study assessing vaccine uptake. Incidence and severity of TBE between 2005 and 2022 were determined using data from the Swiss disease surveillance system and VE was calculated using a case–control analysis, matching TBE cases with SNVCS controls.

**Results:**

Over the study period, vaccination coverage increased substantially, from 4.8% (95% confidence interval (CI): 4.1–5.5%) to 50.1% (95% CI: 48.3–52.0%). Reported clinical symptoms in TBE cases were similar irrespective of age. Neurological involvement was less likely in incompletely (1–2 doses) and completely (≥ 3 doses) vaccinated cases compared with unvaccinated ones. For incomplete vaccination, VE was 66.2% (95% CI: 42.3–80.2), whereas VE for complete vaccination was 90.8% (95% CI: 87.7–96.4). Vaccine effectiveness remained high, 83.9% (95% CI: 69.0–91.7) up to 10 years since last vaccination.

**Conclusions:**

Even children younger than 5 years can experience severe TBE. Incomplete and complete vaccination protect against neurological manifestations of the disease. Complete vaccination offers durable protection up to 10 years against TBE.

**Key public health message**

**What did you want to address in this study and why?**

Tick-borne encephalitis (TBE) is an emerging viral infection of the central nervous system, which is preventable by vaccination. Here, we set out to describe the severity of the disease in children and adolescents in Switzerland and evaluated how effectively the vaccine prevents disease.

**What have we learnt from this study?**

We found that children aged 5 years and younger experience severe disease, like older children and adolescents; something which was not previously well-known. The vaccine was over 66% effective in preventing disease if individuals received one or two doses and over 90% effective if individuals received three or more doses. Effectiveness remained high even up to 10 years since last vaccination.

**What are the implications of your findings for public health?**

As children and adolescents can experience severe TBE, they should be vaccinated if living in or visiting areas where TBE virus is common. Complete vaccination with three or more vaccine doses offers durable protection against disease, suggesting that 10-year booster intervals might be considered for this age group.

## Introduction

Tick-borne encephalitis (TBE) is a severe viral infection of the central nervous system caused by the tick-borne encephalitis virus (TBEV), which is transmitted by Ixodid ticks. In severe cases, the infection can lead to permanent neurological injury and death. Compared with adults, clinical symptoms of TBE are often milder in children [1], although severe disease and deaths can occur. Increasing evidence points towards an underdiagnosis of childhood TBE due to less specific symptoms [1], and recent studies have demonstrated that even mild cases of TBE in childhood can lead to lasting cognitive impairments [2,3]. For those living in or travelling to highly endemic areas, with an annual TBE incidence of ≥ 5.0 cases per 100,000 individuals, the World Health Organization (WHO) recommends vaccination beginning at 1 year of age [4,5]. Since 2006, TBE vaccination in Switzerland has been recommended in risk areas [6] from 6 years of age and reimbursed by compulsory health insurance [7]. Nearly all areas of the country are now considered risk areas [8].

Two vaccines against TBE, administered as a series of three primary doses, are currently licensed in western Europe and available in Switzerland. They are generally considered interchangeable in terms of immunogenicity and durability of humoral immune responses [9]. Vaccination is highly protective against TBE, stimulating virus-neutralising antibodies which are associated with disease prevention, though a formal correlate of protection has not been defined. Vaccine effectiveness (VE) is a measure of how well a vaccine protects immunised individuals against health outcomes. As with many vaccines, TBE vaccine responsiveness and VE are potentially influenced by several factors, including age [10,11] and compliance to the recommended vaccination schedule [12-15]. In Switzerland, booster doses for TBE are given every 10 years after completion of the three dose primary series [7,16], compared with every 3–5 years in most other countries. For adults in Switzerland, we recently reported that VE for incomplete vaccination (1–2 doses) was 77%, while VE for complete vaccination (≥ 3 doses) was 95% and durable, even among those aged ≥ 65 years, for at least 10 years after last vaccination [17]. This finding is consistent with other recent studies in Germany and Latvia [18,19]. Although it now seems clear that VE in adults is robust and long-lasting, less is known about VE in children, particularly regarding the duration of protection.

Here, we used nationwide vaccination record survey data collected between 2005 and 2022 from children and adolescents aged 2, 8 and 16 years, along with TBE case data from the same period for children and adolescents aged 0–17 years obtained from the Swiss national disease surveillance system. We report TBE vaccination coverage, disease severity and VE, along with estimates of disease risk among unvaccinated individuals and number of cases prevented.

## Methods

### Description of vaccination coverage

The Swiss National Vaccination Coverage Survey (SNVCS) is a nationwide, cross-sectional surveillance established in 1999. It evaluates vaccination coverage in children and adolescents aged 2, 8 and 16 years in collaboration with the Swiss Federal Office of Public Health (FOPH), the Epidemiology, Biostatistics and Prevention Institute at the University of Zurich and all 26 Swiss cantons [20]. Vaccination coverage data are collected at the cantonal level over a 3-year rolling cycle, with approximately one third of Swiss cantons participating each year [21]. In brief, children and adolescents aged 2, 8 or 16 years in the survey year for that canton, are selected by cluster sampling or simple random sampling. The families of selected children are contacted by mail and invited to participate in the survey by submitting a copy of the child’s vaccination record. For each mailing, an initial letter and one to two reminder letters requesting participation are sent to each family. In some cases, a final telephone contact is attempted in lieu of a third letter. Dates of vaccination were manually extracted from submitted vaccination records and recorded into the SNVCS database. All survey participants from the six most recent survey periods (2005–2007, 2008–2010, 2011–2013, 2014–2016, 2017–2019 and 2020–2022) were included in this study (n = 123,875). Data for TBE vaccination were analysed using svyset commands for Stata v.17.0 (StataCorp, LLC, College Station, United States (US)), adjusting for sampling method and response and post-stratifying for sex, nationality and urbanisation for each canton (i.e. stratum).

### Description of cases of tick-borne encephalitis

**Figure 1:**
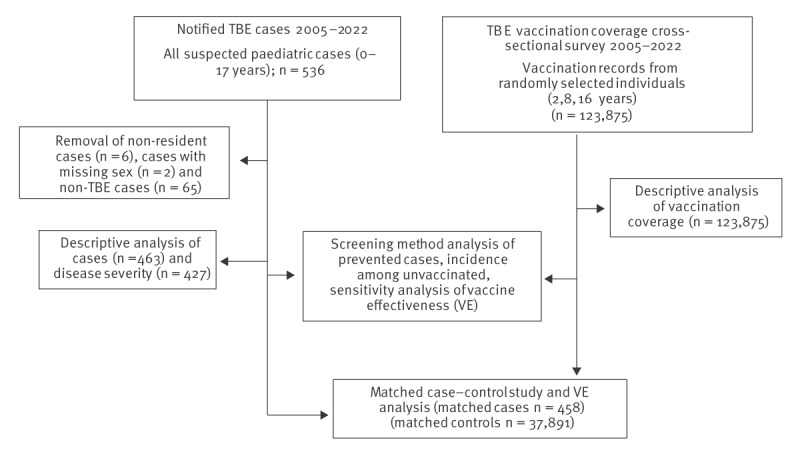
Flowchart of the study evaluating tick-borne encephalitis vaccination coverage, disease severity and vaccine effectiveness in children and adolescents, Switzerland, 2005–2022

TBE: tick-borne encephalitis; VE: vaccine effectiveness.

Tick-borne encephalitis has been a mandatory notifiable disease in Switzerland since 1988, with all suspected cases notified via the national disease surveillance system [22,23]. All diagnostic serological tests positive for TBE are notified by laboratories to the FOPH. Information on clinical diagnosis and vaccination status (number of doses received and date of last vaccination) are then obtained from the responsible physician. The TBE case definition used in Switzerland is similar to, but differs slightly from, that of the European Centre for Disease Prevention and Control (ECDC). In Switzerland, possible cases, which include non-specific neurological or influenza-like symptoms in combination with appropriate serology, in addition to probable and confirmed cases, are included [24-27]. From the FOPH, we obtained data for all suspected TBE cases among individuals aged 0–17 years notified between 2005 and 2022 (n = 536). Cases among non-residents of Switzerland (n = 6) and cases with missing sex information (n = 2) were excluded. From all reports, possible (n = 78), probable (n = 157) and confirmed (n = 228) TBE cases, based on the Swiss TBE case definitions, were included and non-cases (n = 65) were excluded (final n = 463) (Figure 1). Descriptive statistics for cases were calculated by age group (intervals: 0–5, 6–11, 12–17 years). Among the cases with information on clinical diagnoses (n = 427), symptoms were categorised as (i) no neurological involvement or other: symptoms classified as influenza-like, including febrile illness, headache, fatigue and malaise or undefined with no specific symptoms reported, (ii) mild neurological disease: meningeal disease including meningeal irritation or meningitis or (iii) moderate to severe neurological disease: encephalitic disease including meningoencephalitis, encephalomyelitis, radiculitis or paresis [28], and summarised descriptively. The likelihood of being unvaccinated against TBE based on disease symptoms (no neurological involvement or other, mild neurological disease, moderate to severe neurological disease) was evaluated using multiple logistic and multinomial regression modelling, adjusting for sex and age, with odds ratios (ORs) and adjusted ORs (aORs) and relative risk ratios (RRRs) and adjusted RRRs (aRRRs) reported.

### Determination of vaccine effectiveness against tick-borne encephalitis by case–control study

To assess VE, we matched TBE cases to community controls selected from SNVCS participants. Based on the relative rarity of both TBE cases (estimated incidence 2.44/100,000 individuals) and SNVCS study participation (3,650/100,000 individuals), we determined it was unlikely (0.09/100,000 individuals, i.e. the likelihood of being a case multiplied by the likelihood being a control) that any given individual would be both a case and control. Cases and controls were further matched on sex, age (intervals: 0–5, 6–11, 12–17 years), year of case notification or survey participation (intervals: 2005–2007, 2008–2010, 2011–2013, 2014–2016, 2017–2019, 2020–2022) and canton of residence (half-cantons were combined). All possible numbers of matches per each case were considered. Matching was performed using the coarsened exact matching (CEM) function in Stata v.17.0 [29]. A total of 458 cases were matched to 37,891 controls (Figure 1). For both cases and controls, the total number of TBE vaccine doses received and the time since most recent vaccination were determined. Individuals were classified as being unvaccinated (no doses), incomplete (1–2 doses) or complete (≥ 3 doses). Among those with complete vaccination, individuals were further classified by the time since the last dose was received (< 5 years or 5–10 years). In a small subset of controls (0.65%, n = 245), time since the last dose was received exceeded 10 years (maximum 15 years) but was set to 10 years for the analysis. Based on these criteria, a conditional logistic regression model was used to calculate VE using the formula VE = 100 × [1 − OR], where OR is the OR for each of the defined vaccination status categories with disease status as the outcome.

### Estimation of disease incidence among unvaccinated, prevented cases, potentially prevented cases and sensitivity analysis of vaccine effectiveness

The incidence of TBE among unvaccinated individuals (IUV) was calculated using the formula IUV = [(CTOT × FUV) / PUV], where CTOT is the total number of TBE cases, FUV the fraction of TBE cases among unvaccinated individuals determined from FOPH cases with available vaccination record data (n = 388), PUV the unvaccinated population determined from the Swiss Federal Statistical Office population data (SFSO) [30] and SNVCS vaccination coverage data. The incidence of TBE among completely vaccinated individuals (IVC) was calculated using the formula IVC = [(CTOT × FCV) / PVC], where FVC is the fraction of TBE cases among individuals with three or more TBE vaccine doses and PVC the completely vaccinated population. The number of TBE cases prevented by vaccination (prevented cases (PC)) was estimated using the formula PC = [(CTOT × FVC) − (PVC × IUV)]. The number of TBE cases potentially prevented, had the entire population been vaccinated (potentially prevented cases (PPC)), was estimated using the formula PPC = [CTOT × (((CTOT / P) − IVC) / (CTOT / P))], where P is the total population. All values were calculated for each 3-year SNVCS survey period, as well as for the overall period between 2005 and 2022. As a sensitivity analysis, we additionally calculated VE for the most recent SNVCS survey period (2020–2022) using the screening method [31,32], using the formula VE = [1 − (IVC / IUV)].

### Statistical analyses

Descriptive statistics, case–control matching and regression analyses were performed using Stata v.17.0 (StataCorp LLC, the United States (US)) and visualised using GraphPad Prism v.10.0 (GraphPad Software, Inc., San Diego, US). For all assessments, p values < 0.05 were considered statistically significant.

## Results

### Vaccination coverage for tick-borne encephalitis at the national level by age

**Figure 2:**
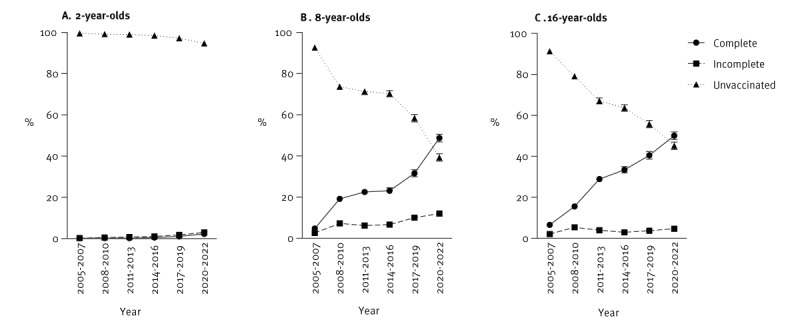
Tick-borne encephalitis vaccination coverage of children and adolescents, by age, Switzerland, 2005–2022 (n = 123,875)

Data for TBE vaccination from the SNVCS survey for 123,875 children and adolescents across Switzerland aged 2, 8 and 16 years were included in this study (Figure 1, Table 1). Overall, 51.2% (95% confidence interval (CI): 50.8–51.6%) of participants were male, 33.2% (95% CI: 32.9–33.6%) were aged 2 years, 32.5% (95% CI: 32.1–32.9%) were aged 8 years and 34.3% (95% CI: 33.9–34.6%) were aged 16 years (Table 1). The median age of first vaccination was 7 years (Interquartile Range (IQR): 6–9 years). We found that, since the initial 2006 recommendation for TBE vaccination of individuals aged ≥ 6 years residing in risk areas, vaccination progressively increased among those aged 8 and 16 years in the population over time, with complete vaccination (≥ 3 doses) increasing from 4.8% (95% CI: 4.1–5.5%) in the 2005–2007 survey period to 48.7% (95% CI: 46.9–50.6%) in 2020–2022 for 8-year-olds and from 6.6% (95% CI: 5.6–7.3%) in 2005–2007 to 50.1% (95% CI: 48.3–52.0%) in 2020–2022 for 16-year-olds (Figure 2). In accordance with the vaccination recommendation, coverage remained low among 2-year-olds: 0.1% (95% CI: 0.07–0.2%) in the 2005–2007 survey period and 2.3% (95% CI: 1.8–2.9%) in the 2020–2022 survey period (Figure 2)

**Table 1:** Characteristics of Swiss National Vaccination Coverage Survey participants (n = 123,875) and tick-borne encephalitis cases (n = 463) of a study evaluating tick-borne encephalitis vaccination coverage, disease severity and vaccine effectiveness in children and adolescents, Switzerland, 2005–2022

Participants	2005–2007		2008–2010		2011–2013		2014–2016		2017–2019		2020–2022		Overall	
SNVCS study participants^a^ (n = 123,875)														
Participation rate	83.0%		79.8%		77.7%		69.6%		66.0%		57.6%		72.3%	
Characteristics	%	95% CI	%	95% CI	%	95% CI	%	95% CI	%	95% CI	%	95% CI	%	95% CI
Sex														
Male	51.0	50.3–51.9	51.4	50.6–52.3	51.1	50.2–52.0	51.0	50.1–52.0	51.3	50.3–52.4	51.2	50.1–52.3	51.2	50.8–51.6
Female	48.9	48.1–49.7	48.6	47.7–49.4	48.9	48.0–49.8	49.0	48.0–50.0	48.7	47.7–49.7	48.8	47.7–49.9	48.8	48.4–49.2
Age (years)														
2	27.7	27.1–28.4	31.4	30.6–32.1	33.8	33.0–34.7	34.0	33.0–34.9	36.7	35.7–37.7	35.7	34.7–36.7	33.2	32.9–33.6
8	34.4	33.7–35.2	31.7	30.9–32.5	31.1	30.3–31.9	32.4	31.5–33.3	32.2	31.3–33.2	33.1	32.1–34.1	32.5	32.1–32.9
16	37.9	37.1–38.7	36.9	36.1–37.8	35.1	34.2–36.0	33.6	32.7–34.6	31.1	30.2–32.1	31.2	30.3–32.2	34.3	33.9–34.6
Vaccination status														
Incomplete	1.8	1.6–2.0	4.4	4.1–4.8	3.6	3.3–4.0	3.5	3.1–3.8	5.1	4.6–5.5	6.5	6.0–7.1	4.2	4.0–4.3
Complete	4.2	3.8–4.6	11.9	11.4–12.5	17.2	16.6–17.9	18.9	18.2–19.7	23.2	22.3–24.3	32.6	31.6–33.6	18.2	17.9–18.5
Unvaccinated	94.1	93.6–94.5	83.7	83.0–84.3	79.2	78.5–79.9	77.6	76.8–78.4	71.8	70.8–72.7	60.9	59.9–62.0	77.7	77.3–78.0
TBE cases (n = 463)														
Characteristics	n	%	n	%	n	%	n	%	n	%	n	%	n	%
Total	101	21.8	33	7.1	69	14.9	43	9.3	105	22.7	112	24.2	463	100.0
Sex														
Male	63	62.4	20	60.6	48	69.6	30	69.8	65	61.9	70	62.5	296	63.9
Female	38	37.6	13	39.4	21	30.4	13	30.2	40	38.1	42	37.5	167	36.1
Age (years)														
0–5	13	12.9	6	18.2	13	18.8	10	23.3	31	26.5	27	24.1	100	21.6
6–11	50	49.5	13	39.4	25	36.2	18	41.9	38	36.2	44	39.3	188	40.6
12–17	38	37.6	14	42.4	31	44.9	15	34.9	36	34.3	41	36.6	175	37.8
Vaccination status (n = 388)														
Total	78	20.1	28	7.2	62	16.0	34	8.8	94	24.2	92	23.7	388	100.0
Incomplete	3	3.9	0	0.0	5	8.1	2	5.9	3	3.2	4	4.4	17	4.4
Complete	2	2.6	2	7.1	5	8.1	3	8.8	2	2.1	9	9.8	23	5.9
Unvaccinated	73	93.6	26	92.9	52	83.9	29	85.3	89	94.7	79	85.9	348	89.7
Symptoms (n = 427)														
Total	96	22.5	33	7.7	66	15.5	39	9.1	97	22.7	96	22.5	427	100.0
Non-neurological influenza-like	5	5.2	3	9.1	5	7.6	1	2.6	12	12.4	9	9.4	35	8.2
Non-neurological, undefined	5	5.2	2	6.1	5	7.6	3	7.7	3	3.1	3	3.1	21	4.9
Mild neurological	50	52.1	9	27.3	24	36.4	9	23.1	28	28.9	29	30.2	149	34.9
Moderate neurological	33	34.4	18	54.6	30	45.5	26	66.7	52	53.6	50	52.1	209	49.0
Severe neurological	3	3.1	1	3.0	2	3.0	0	0.0	2	2.1	5	5.2	13	3.0

CI: confidence interval; SNVCS: Swiss National Vaccination Coverage Survey; TBE: tick-borne encephalitis.

^a^ As survey data are weighted, numbers of participants per characteristic are not included.

Complete vaccination: ≥ 3 vaccine doses; Incomplete vaccination: 1-2 vaccine doses; Unvaccinated: no TBE vaccine doses.

CI: confidence interval.

Complete vaccination: ≥ 3 vaccine doses; Incomplete vaccination: 1-2 vaccine doses; Unvaccinated: no TBE vaccine doses.

Percentages of complete vaccination (≥ 3 doses) for 2-year-olds: 0.1% (95% CI: 0.07–0.2%) in the 2005–2007 survey period and 2.3% (95%CI: 1.8–2.9%) in the 2020–2022 survey period; for 8-year-olds: 4.8% (95% CI: 4.1–5.5%) in the 2005–2007 survey period and 48.7% (95% CI: 46.9–50.6%) in 2020–2022; for 16-year-olds: 6.6% (95% CI: 5.6–7.3%) in 2005–2007 and 50.1% (95% CI: 48.3–52.0%) in the 2020–2022 survey period.

We additionally observed that overall vaccination coverage in the study population varied widely by canton, ranging from 1.1% (95% CI: 0.5–2.4%) in Geneva (one of only two cantons where vaccination is not recommended) to 46.5% (95% CI: 43.1–50.0%) in Zurich in the most recent (2020–2022) survey period, as can be seen in Supplementary Figure 1. The incidence of TBE is not uniform and has progressively spread from the north-east of the country over time [4,33], impacting risk area definitions and corresponding vaccination recommendations. We considered the influence of average disease incidence in the canton of residence between 2005 and 2022 [4] on having received at least one TBE vaccine dose. Notably, we observed moderate to very strong correlations between TBE vaccination and average disease incidence in the canton of residence in each survey period, as presented in Supplementary Figure 2, with each additional unit increase in average annual TBE incidence (1 case/100,000 individuals) associated with a 1.3-fold increased likelihood of being vaccinated (OR = 1.30; 95% CI: 1.29–1.31), as presented in Supplementary Table 1. Together, these findings demonstrate an increase in TBE vaccine uptake among children and adolescents over time, corresponding to the official recommendation for vaccination in 2006 and correlating with disease incidence in the canton of residence.

### Cases of tick-borne encephalitis and disease severity among children and adolescents

**Figure 3:**
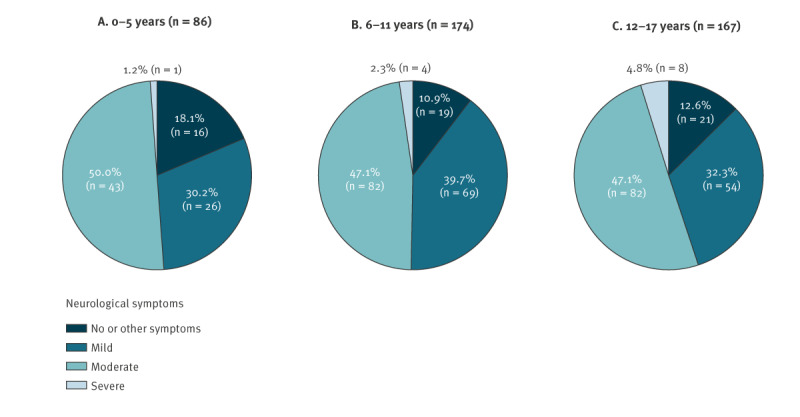
Notified cases of tick-borne encephalitis in children and adolescents, by age and disease severity, Switzerland, 2005–2022 (n = 427)

Of the 463 TBE cases included in the study, 296 (63.9%) were males, 100 (21.6%) were aged 0–5 years (incidence/100,000: 1.87; 95% CI: 1.50–2.25), 188 (40.6%) were aged 6–11 years (incidence/100,000: 2.87; 95% CI: 2.45–3.29) and 175 (37.8%) were aged 12–17 years (incidence/100,000: 2.46; 95% CI: 2.09–2.83) (Table 1). The median age was 10 years (IQR: 6–14 years). Among the TBE cases with information on clinical symptoms (n = 427), 56 (13.1%) had no neurological involvement. Of these 56, 35 had influenza-like symptoms, with headache, fever, fatigue and nausea most commonly reported. Mild neurological disease (meningeal irritation and meningitis) was observed in 149 (34.9%) cases, moderate disease (meningoencephalitis) in 209 (49.0%) cases and severe disease (encephalitis, encephalomyelitis, radiculitis, paresis) was reported in 13 (3.0%) cases. The breakdown of disease severity was similar across the three age groups (Figure 3).

Mild neurological disease: meningeal irritation, meningitis; Moderate disease: meningoencephalitis; Severe disease: encephalitis, encephalomyelitis, radiculitis, paresis.

We further considered the relationship between disease severity and vaccination status in vaccination breakthrough infection (Table 2). We found that unvaccinated individuals were substantially more likely than vaccinated individuals to have neurological disease symptoms (mild vs non-neurological or other symptoms: aOR = 6.7; 95% CI: 2.1–21.7). We also observed similar results stratifying by incomplete and complete vaccination. Incompletely vaccinated cases were 84% less likely to experience mild neurological disease compared with unvaccinated cases (aRRR = 0.16; 95% CI: 0.03–0.93) and completely vaccinated were 86% less likely to do so compared with unvaccinated cases (aRRR = 0.14; 95% CI: 0.03–0.63). No significant results were seen between incomplete and complete vaccination and reduced risk of moderate and severe disease compared with unvaccinated individuals. Importantly, these findings suggest that vaccination is protective against disease, especially against the development of mild neurological disease.

**Table 2:** Association between disease severity and vaccination status of notified cases of tick-borne encephalitis in children and adolescents, by age and sex and results of regression analyses, Switzerland, 2005–2022 (n = 427)

Characteristics	Univariate			Multivariate		
	OR	95% CI	p value	aOR	95% CI	p value
Logistic regression: unvaccinated vs vaccination with ≥ 1 vaccine doses						
Sex						
Female	Reference					
Male	1.01	0.51–1.98	0.986	0.85	0.41–1.78	0.670
Age						
Each additional year	0.89	0.82–0.97	0.007	0.88	0.80–0.96	0.003
Disease severity						
Non-neurological symptoms	Reference					
Mild neurological symptoms	5.81	1.88–17.97	0.002	6.71	2.08–21.71	0.001
Moderate or severe neurological symptoms	1.75	0.77–3.96	0.179	1.98	0.84–4.70	0.119
Characteristics	RRR	95% CI	p value	aRRR	95% CI	p value
Multinomial regression: unvaccinated vs incomplete vs complete vaccination						
Unvaccinated	Reference					
Incomplete Vaccination						
Sex						
Female	Reference					
Male	1.09	0.39–3.02	0.864	1.16	0.39–3.41	0.790
Age						
Each additional year	1.02	0.91–1.15	0.681	1.05	0.93–1.18	0.456
Disease severity						
Non-neurological symptoms	Reference					
Mild neurological symptoms	0.17	0.03–0.97	0.047	0.16	0.03–0.93	0.042
Moderate or severe neurological symptoms	0.62	0.19–1.50	0.440	0.58	0.17–2.02	0.396
Complete vaccination						
Sex						
Female	Reference					
Male	0.93	0.39–2.20	0.865	1.18	0.46–3.06	0.730
Age						
Each additional year	1.21	1.07–1.35	0.001	1.23	1.09–1.40	0.001
Disease severity						
Non-neurological symptoms	Reference					
Mild neurological symptoms	0.17	0.04–0.72	0.016	0.14	0.03–0.63	0.010
Moderate or severe neurological symptoms	0.54	0.19–1.50	0.236	0.44	0.15–1.34	0.149

aOR: adjusted odds ratio; aRRR: adjusted relative risk ratio; CI: confidence interval; OR: odds ratio; RRR: relative risk ratio.

### Vaccine effectiveness, cases prevented and potentially prevented due to vaccination and incidence in the unvaccinated population

**Figure 4:**
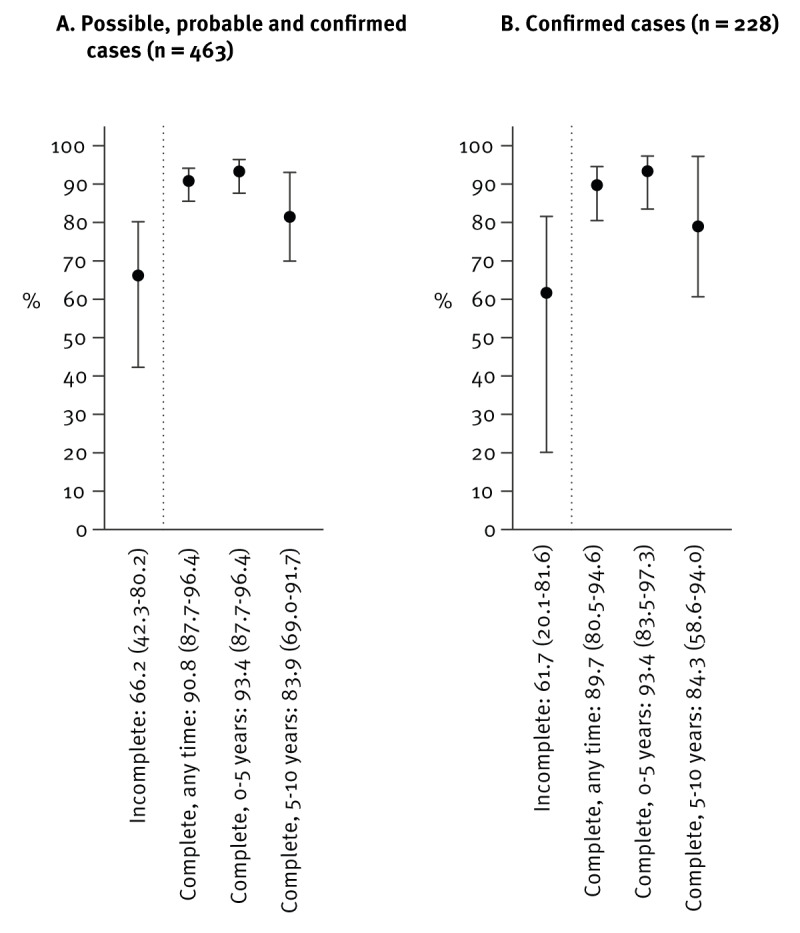
Vaccine effectiveness against tick-borne encephalitis in children and adolescents, by vaccination status and time, Switzerland, 2005–2022 (n = 463)

We then evaluated TBE VE (against all disease severity), matching notified TBE cases to community controls from the SNVCS database. A total of 458 cases were matched to 37,891 controls (Figure 1). We found that VE for incomplete vaccination was 66.2% (95% CI: 42.3–80.2) (Figure 4A). The median time between last vaccination and onset of illness for cases was less than 1 year (range: 0–16 years) while the median time since last vaccination for controls was 1 year (range: 0-16 years), see more details in Supplementary Table 2. Overall, VE for complete vaccination was 90.8% (95% CI: 87.7–96.4). Median time between last vaccination and onset of illness for cases was 5.5 years (range: 0–9 years) while the median time since last vaccination for controls was 3 years (range: 0–15 years). For complete vaccination within the past 5 years, VE was 93.4% (95% CI: 87.7–96.4), while VE for complete vaccination 5–10 years earlier was 83.9% (95% CI: 69.0–91.7) (Figure 4A) and Supplementary Table 2. We obtained similar results considering only confirmed TBE cases (Figure 4B). As a sensitivity analysis, we used the screening method [31], as an additional means to estimate VE. Using data from the five most recent SNVCS survey periods (2008–2022), adjusting for the change in TBE vaccination recommendation in 2006, which interrupted the 2005–2007 survey period, we found that the overall VE for complete vaccination (≥ 3 doses) was 72.8% (95% CI: 59.1–81.9). As vaccination rates varied substantially by age group, we further stratified by those aged 0–5, 6–11 and 12–17 years. For 0–5-year-olds, VE was 100%, for 6–11-year-olds it was 86.3% (95% CI: 72.6–93.1) and for 12–17-year-olds VE was 72.7% (95% CI: 54.6–83.6), differing somewhat from, but still consistent with, our estimate from the case–control analysis, presented in Supplementary Table 3.

Complete vaccination: ≥ 3 vaccine doses; Incomplete vaccination: 1-2 vaccine doses; Unvaccinated: no TBE vaccine doses.

Confirmed: clinical criteria: any person with symptoms of inflammation of the central nervous system (for example, meningitis, meningoencephalitis, encephalomyelitis, encephaloradiculitis) and at least one of the following laboratory criteria: TBE specific IgM and IgG antibodies in blood, TBE specific IgM antibodies in cerebrospinal fluid, seroconversion or four-fold increase of TBE-specific antibodies in paired serum samples, detection of TBE viral nucleic acid in a clinical specimen, isolation of TBE virus from clinical specimen.

Possible: non-specific neurological or influenza-like symptoms in combination with appropriate serology.

Probable: any person meeting the clinical criteria and detection of TBE-specific IgM-antibodies in a unique serum sample or any person meeting the clinical criteria with an epidemiological link.

We further estimated the incidence among unvaccinated (IUV) for each of the six 3-year SNVCS survey periods between 2005 and 2022. The IUV increased over time, most strikingly since 2017, peaking in the most recent survey period (2020–2022) at 4.9 (95% CI: 4.1–5.6) cases per 100,000 individuals aged 0–17 years, as presented in Supplementary Figure 3A. In contrast, the incidence among vaccinated individuals (IV) remained near or below 1.0 case per 100,000 individuals since 2007 (1.0/100,000; 95% CI: 0.68–1.4 in the 2020–2022 survey period), presented in Supplementary Figure 3A. Comparing the IUV and IV, the risk of disease between 2005 and 2022 was, on average, 3.6 (95% CI: 2.3–5.4) fold higher among unvaccinated individuals compared with vaccinated, with a peak fold-increase in risk of 14.4 (95% CI: 4.1–50.4) in the 2017–2019 survey period, as shown in Supplementary Figure 3B. We further estimated the number of cases prevented by TBE vaccination and the number of additional cases that could have been prevented had the entire population aged 0–17 years been vaccinated. We found that the number of TBE cases prevented by vaccination increased over time, with 41 (95% CI: 29–55) cases prevented in the 2020–2022 survey period, shown in Supplementary Figure 3C. In total, we estimate that vaccination prevented 70 (95% CI: 54–88) TBE cases among children and adolescents aged 0–17 years between 2005 and 2022, Supplementary Figure 3C. During this same time, an additional 312 (95% CI: 278–348) TBE cases could potentially have been prevented had all individuals aged 0–17 years been completely vaccinated, Supplementary Figure 3C. An estimated 100 (95% CI: 81–122) of these prevented cases would have been among children aged 0–5 years.

## Discussion

An increased awareness of nationwide patterns in TBE vaccination and uptake, along with a better understanding of the level and durability of VE, can provide valuable information to support vaccination recommendations and identify areas for improvement. This is particularly important in light of the rapidly changing epidemiology of this disease [34].

We recently reported that, among adults aged 18–79 years in Switzerland, 34% had been completely vaccinated (≥ 3 doses) against TBE, with significant variation between regions based on differences in disease incidence and vaccination recommendations [35]. Using data from the SNVCS survey, which routinely assesses vaccination coverage among children aged 2, 8 and 16 years throughout the country, we found that, since the 2006 recommendation for TBE vaccination of those aged ≥ 6 years residing in risk areas, overall TBE vaccination coverage has increased. While vaccination coverage (both complete and incomplete) has remained low among children aged 2 years, which is not surprising as the current vaccination recommendation is from the age of 6 years, coverage among those aged 8 and 16 years has increased (10- and 7.5-fold for complete coverage, respectively), with just over 50% of the 16-year-olds nationwide being completely vaccinated. Like adults [35], vaccination coverage varies widely throughout the country, although it correlates well with the average annual TBE incidence in the canton of residence. Prior to a 2019 change in vaccination recommendations, which now advises TBE vaccination for all individuals from 6 years of age throughout the country except for two cantons (Geneva and Ticino) [8], TBE vaccination was recommended based on annual risk assessments by the FOPH. Between 2006 and early 2013, risk areas included locations with at least three notified TBE cases or detection of TBEV in local tick populations. From April 2013 onwards, risk areas have been defined as locations with a more than expected number of cases based on historical data using a standardised algorithm [6]. Recommendations were updated annually to account for the geographical spread of the virus and increase in TBE incidence over time [6,16,36]. Therefore, average incidence has previously played an important role in determining areas in which TBE vaccination was recommended.

Compared with adults, clinical symptoms of TBE are generally considered milder in children [1], particularly in young children. Although fewer cases of TBE were observed in the youngest age group of 0–5 years, the average disease incidence over time did not differ substantially between this group and those aged 6–17 years. That there were also similarities between the overall breakdown of clinical diagnoses between age groups suggests that even young children remain at risk of TBE, including severe disease. Among TBE cases, we further found that the odds of a meningitis diagnosis were substantially increased (compared to having no or questionable neurological symptoms) among unvaccinated individuals, consistent with the protective role of vaccination. While controversial, it has been speculated that TBE vaccination breakthrough infections might be associated with a more severe disease course compared with unvaccinated individuals [13,15,37-39]. Our findings, however, do not support this. It is worth noting that even incomplete vaccination appeared to convey a reduced risk of neurological illness, which is important to consider for travellers, who may not have sufficient time to complete a three-dose primary series before exposure.

Although vaccination breakthrough cases were uncommon, among incompletely vaccinated individuals, we found that the time to vaccine breakthrough was short – less than 1 year from when the last dose was received. In contrast, vaccine breakthroughs in fully vaccinated individuals (≥ 3 dose recipients) occurred a median of 5.5 years following receipt of the last vaccine dose. We reported similar findings in adults [17]. Together, these findings suggest that individuals may not have had sufficient time to develop protective immunity between vaccination and exposure. It may be prudent to warn individuals to wait a minimum of several weeks following vaccination before any planned exposure or to initiate vaccination in the winter before exposure, together with the influenza vaccination, for example, to ensure sufficient time for completion of the full primary series of three doses.

While most other European countries recommend a TBE vaccine booster interval of 3–5 years depending on age, Switzerland and Finland with some exceptions, recommend boosters every 10 years [7,16,40]. The durability of protection, however, is important to understand for such recommendations. In this study, we found that, among children and adolescents, VE for incomplete vaccination was slightly over 66%, while VE for complete vaccination was 93% for vaccination within the past 5 years and 84% for vaccination completed 5–10 years before, suggesting the prolonged effectiveness of TBE vaccination. Recent studies from both Austria and Latvia have reported similar results [41,42], with VE estimates in children of > 91% and > 99%, respectively. In our recent study of adults in Switzerland, VE for incomplete vaccination was 77% and VE for complete vaccination was 95% and was similar when the most recent dose was received < 5 years, 5–10 years or ≥ 10 years before [17]. Together, these results are consistent with our previous findings in adults and support the continued high effectiveness of TBE vaccination in children, even up to 10 years from the last dose received.

We further assessed the impact of TBE vaccination in children and adolescents, estimating the IUV, which helps to provide an estimate of the true risk of TBE infection. Such information is important for decisions on vaccination recommendations. For example, the WHO recommends TBE vaccination beginning at 1 year of age in highly endemic areas, with a pre-vaccination incidence of 5 per 100,000 individuals or higher [4,5]. This is also relevant for travel medicine to aid in the decision to suggest vaccination for travel to certain areas. While Switzerland maintains detailed, publicly accessible data on average TBE incidence throughout the country [4], pre-vaccination incidence is not clear. Here we found that, among individuals aged 0–17 years, IUV reached 4.9 cases per 100,000 in the most recent survey period (2020–2022), i.e. just below the WHO’s highly endemic threshold. Over the entire 18-year study period, the risk of TBE among unvaccinated was nearly 3.6 times higher than among vaccinated individuals in this age group. The continued increase in IUV, despite increasing vaccination coverage, highlights the growing risk of TBE in Switzerland. We estimated that TBE vaccination prevented 70 cases of disease among individuals aged 0–17 years over the study period and could have potentially prevented 312 cases during this period had the entire study population been fully vaccinated. Not surprisingly, the number of prevented cases increased over time as vaccination coverage increased. As there are currently no interventions to control the spread of the TBE virus in the environment, personal protective measures, including vaccination, remain the only means of disease control.

Some limitations of our study include the relatively small number of vaccinated cases (n = 40) over the study period. This resulted in relatively large confidence intervals, particularly in our analyses of the individual 3-year SNVCS survey periods. Furthermore, we assessed clinical disease categorically, which does not account for nuances in disease severity. In addition, clinical information was limited for some cases and, in others, not specified at all. We did not have data on other factors which might impact the response to vaccination and, thus, could not control for them in our analysis. Knowing whether vaccine breakthrough cases have a chronic medical condition or immunosuppression [43-46], or whether they were regularly vaccinated in their primary series [10,11], for example, could potentially influence our assessment of VE. Participation in the SNVCS is high (72% overall), but has decreased over time. It is possible, for example, that non-participants are more or less likely to be vaccinated for TBE. This could, in turn, lead to over or underestimation of VE, respectively.

## Conclusion

Taken together, we find a continued increase in vaccine uptake over time among children and adolescents in Switzerland, consistent with the expansion of the FOPH recommendations. For incomplete vaccination, VE was nearly 70% and exceeded 90% for complete vaccination. For completely vaccinated individuals, VE remained high, even up to 10 years from the last vaccination. Reported clinical symptoms appeared similar between children younger than 5 years and those aged 6–17 years. Together, these findings suggest that TBE vaccination is effective for periods up to 10 years post-vaccination in children and adolescents and that it is highly protective against the development of neurological symptomatic disease.

## Supplementary Data

3513000 Supplementary Material
