# Addendum for Euro Surveill. 2024;29(18)

**DOI:** 10.2807/1560-7917.ES.2024.29.21.240523A

**Published:** 2024-05-23

**Authors:** Eurosurveillance editorial team

**Affiliations:** 1 European Centre for Disease Prevention and Control (ECDC), Stockholm, Sweden

In the article ‘A combined cross-sectional analysis and case–control study evaluating tick-borne encephalitis vaccination coverage, disease and vaccine effectiveness in children and adolescents, Switzerland, 2005 to 2022’ by Zens et al. published in *Eurosurveillance* on 2 May 2024, a Conflict of interest statement was added for authors Kyra D Zens, Robert Steffen and Phung Lang. Moreover, an Acknowledgements section was added, the authors thanking the health departments of all 26 cantons and the Federal Office of Public Health. These additions were made on 23 May 2024.

