# Supplementary material for: A combined cross-sectional analysis and case-control study evaluating tick-borne encephalitis vaccination coverage, disease and vaccine effectiveness in children and adolescents, Switzerland, 2005 to 2022
Source: Euro Surveill. 2024 May 2;29(18):2300558. doi: 10.2807/1560-7917.ES.2024.29.18.2300558 (PMC11067431; doi:10.2807/1560-7917.ES.2024.29.18.2300558)
Supplement: Supplementary Material [file 23-00558_LANG_Supplement.pdf]

This supplementary material is hosted by *Eurosurveillance* as supporting information alongside the article "*A Combined Cross-Sectional Analysis and Case-Control Study Evaluating Tick-Borne Encephalitis Vaccination Coverage, Disease and Vaccine Effectiveness in Children 0-17 in Switzerland, 2005-2022*", on behalf of the authors, who remain responsible for the accuracy and appropriateness of the content. The same standards for ethics, copyright, attributions and permissions as for the article apply. Supplements are not edited by *Eurosurveillance* and the journal is not responsible for the maintenance of any links or email addresses provided therein."

Supplementary Table 1. Factors Influencing Immunization Status at the Population Level

Unimmunized vs. 1+ Dose Vaccination	OR	95% CI	p Value
**Sex**			
**Female**	Ref.	-	-
**Male**	1.09	1.04-1.14	<0.0001
**Age**			
**0-5**	Ref.	-	-
**6-11**	31.23	27.89-34.96	<0.0001
**12-17**	32.28	28.83-36.13	<0.0001
**SNVCS Survey Period**			
**2005-2007**	Ref.	-	-
**2008-2010**	5.37	4.89-5.90	<0.0001
**2011-2013**	6.98	6.37-7.64	<0.0001
**2014-2016**	8.56	7.81-9.39	<0.0001
**2017-2019**	7.68	7.00-8.43	<0.0001
**2020-2022**	12.11	11.03-13.29	<0.0001
**TBE Incidence in Canton of Residence**			
**Each 1.0/100K Increase**	1.30	1.29-1.31	<0.0001

Supplementary Table 2. Time Post-Vaccination for Vaccine Effectiveness (VE) Groups

	Cases			Controls		
Vaccination Status	n (unknown*)	Median (Years)	Range (Years)	n (unknown*)	Median (Years)	Range (Years)
**Incomplete**	15 (2)	0	0-9	1,912 (30)	1	0-16
**Complete (Overall)**	22 (1)	5.5	0-9	8,827 (43)	3	0-15
**Complete (0-5 Years Prior)**	11	2	0-5	6,432	2	0-5
**Complete (5-10 Years Prior)**	11	8	6-9	8,827**	8	6-15**
**Unvaccinated**	345	-	-	27,079	-	-

***Number of individuals with unknown time since last vaccination**

****controls with time since last vaccination exceeding 10 years (n=245) were set to 10 years for the analysis**

Supplementary Table 3. Calculation of Vaccine Effectiveness (VE) using the Screening Method

	Age 0-17	95% CI	Age 0-5	95% CI	Age 6-11	95% CI	Age 12-17	95% CI
**TBE Cases (Ctot)**	362	-	87	-	138	-	137	-
**TBE Cases with known Vaccination Status**	310	-	73	-	120	-	117	-
**Completely Vaccinated Cases (3+ Doses)**	21	-	0	-	7	-	14	-
**Unvaccinated Cases (0 Doses)**	275	-	70	-	108	-	97	-
**Fraction of Cases Completely Vaccinated (Fvc)**	6.8%	-	0.00%	-	5.8%	-	12.0%	-
**Fraction of Cases Unvaccinated (Fuv)**	88.7	-	95.9%	-	90.0%	-	82.9%	-
**Population Size (P)***	15,826,664	-	4,524,761	-	5,450,496	-	5,851,407	-
**Fraction Population Completely Vaccinated (PFvc)**	20.9%	20.5-21.2%	0.90%#	0.76-1.1%	29.4%	28.7-30.1%	33.2%	32.5-35.9%
**Fraction Population Unvaccinated (PFuv)**	74.5%	74.1-74.9%	97.6%	97.4-97.9%	62.1%	61.4-62.8%	62.7%	62.0-63.4%
**Population Completely Vaccinated (Pvc)**	3,306,190	-	40,723	-	1,601,901	-	1,940,912	-
**Population Unvaccinated (Puv)**	11,787,699	-	4,416,619	-	3,385,303	-	3,667,077	-
**Incidence Among Completely Vaccinated (Ivc)****	7.42^06	4.42^06-1.04^05	0.0	0.0-0.0	5.03^06	1.48^06-8.57^06	8.45^06	4.27^06-1.26^06
**Incidence Among Unvaccinated (Iuv)****	2.72^05	2.42^05-3.03^05	1.89^05	1.48^05-2.30^05	3.67^05	3.01^05-4.33^05	3.10^05	2.52^05-3.68^05
**Prevented Cases in Population (PC)§**	65.5	50.6-83.4	0.8	0.0-4.7	50.7	37.5-66.5	43.7	31.5-58.5
**Vaccine Effectiveness (VE)§§**	72.8%	59.1-81.9%	100.0%	-	86.3%	72.6-93.1%	72.7%	54.6-83.6%

***2008-2022 Population data from Swiss Federal Statistical Office**
****Incidence calculated as: Ivc = ((Ctot * Fvc) / Pvc) and Iuv = ((Ctot * Fuv) / Puv)**
§Prevented Cases in Population calculated as: PC = (Ctot * Fvc) - (Pvc * Iuv)
§§Vaccine Effectiveness calculated as: VE = (1 - (Ivc / Iuv)))
#VE calculated from very low vaccination coverage populations should be interpreted with caution

Supplementary Figure 1. Cantonal-Level TBE Vaccination Coverage by SNVCS Survey Period

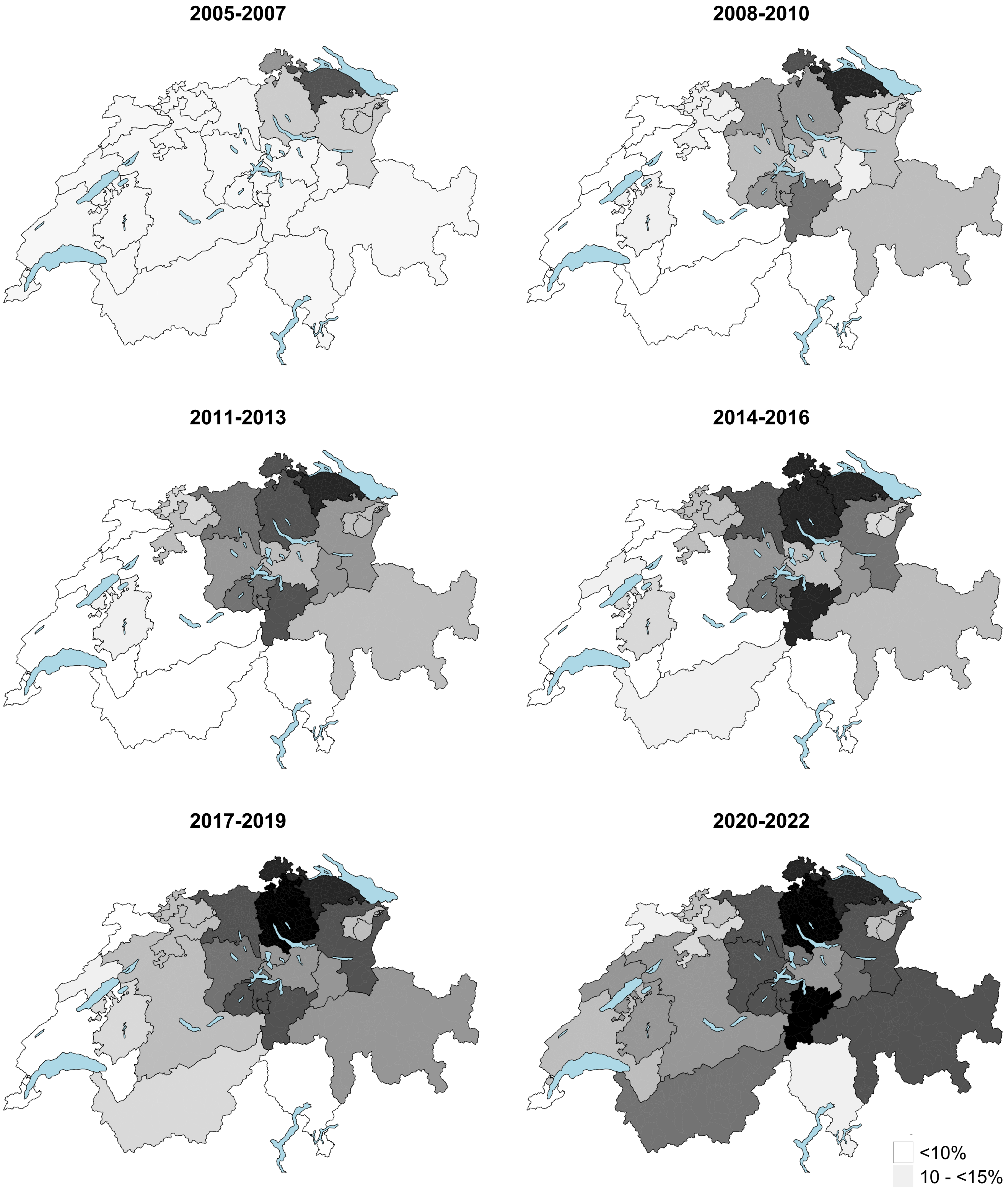

Supplementary Figure 1. Cantonal-Level TBE Vaccination Coverage by SNVCS Survey Period. Average complete (3+ doses) TBE vaccination coverage among 2, 8, and 16 year-olds by Canton of residence and SNVCS survey period.

Supplementary Figure 2. Cantonal-Level TBE Vaccination Coverage by Average TBE Disease Incidence

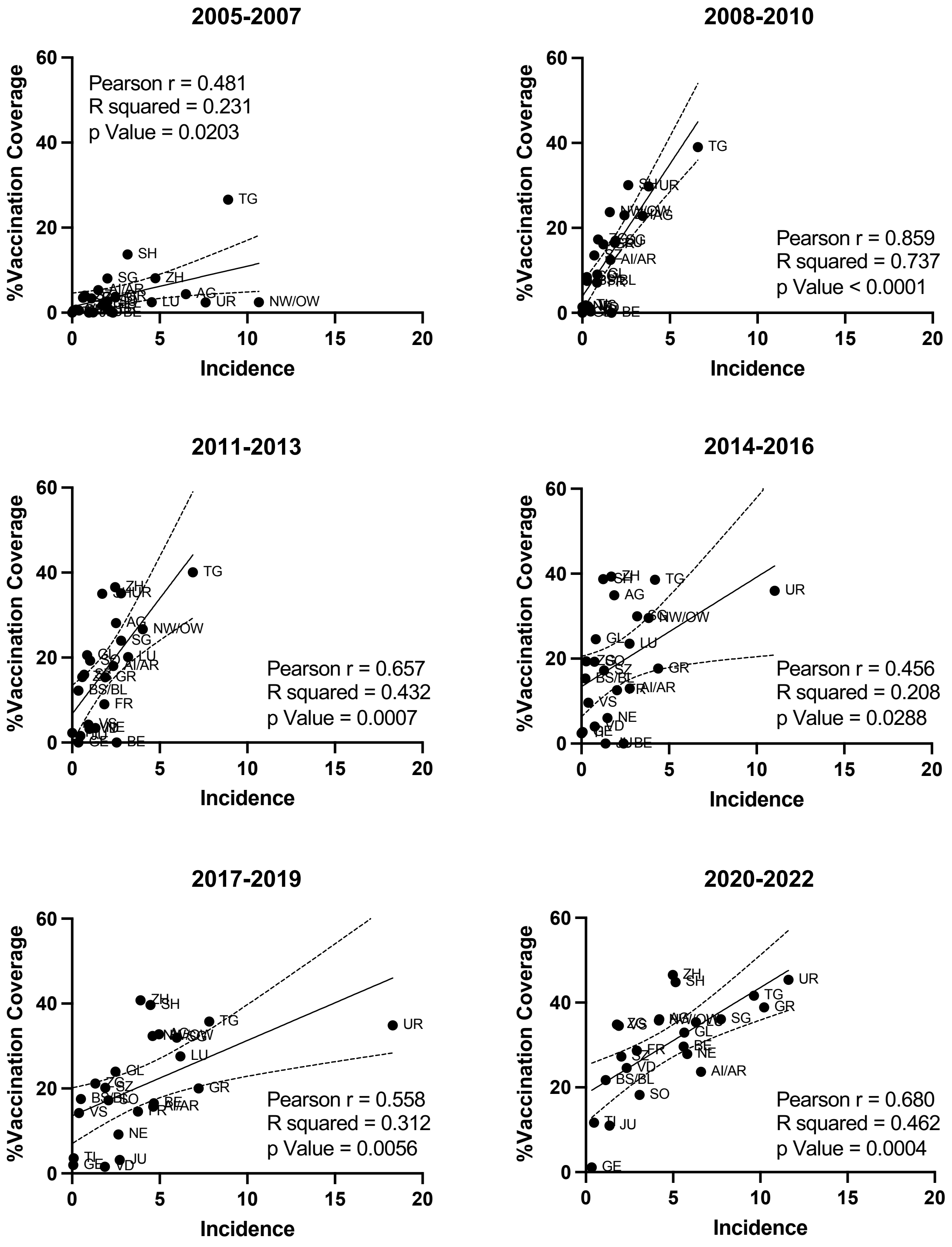

Supplementary Figure 2. Cantonal-Level TBE Vaccination Coverage by Average TBE Disease Incidence. Average complete (3+ doses) TBE vaccination coverage among 2, 8, and 16 year-olds by Canton of residence compared to the average TBE disease incidence in the Canton of residence in the same time period (data from the Swiss FOPH). Pearson correlation coefficients, R squared, and p values for comparisons during each SNVCS survey period are reported. Dotted lines represent 95% confidence bands of the best-fit line.

Supplementary Figure 3. Determination of TBE Incidence in Unvaccinated and Vaccinated, Risk Ratio, and Prevented Cases in Children 0-17 by Survey Period

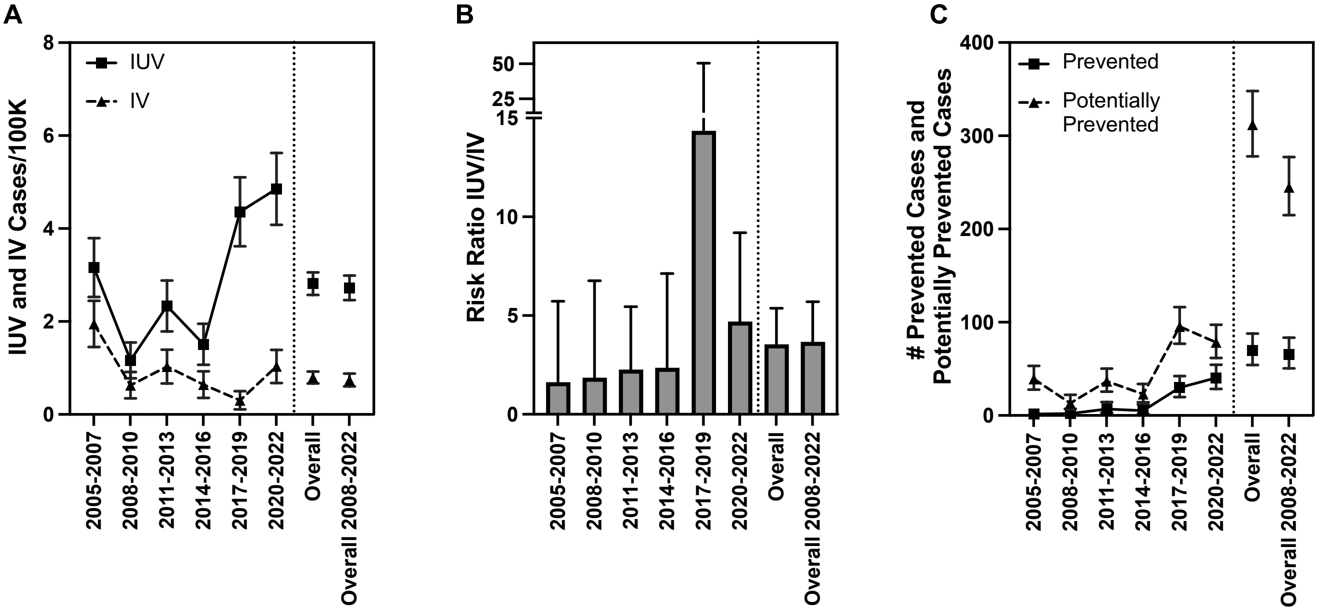

Supplementary Figure 3. Determination of TBE Incidence in Unvaccinated and Vaccinated, Risk Ratio, and Prevented Cases in Children 0-17 by Survey Period. Using the Screening Method, (A) TBE Incidence in Unvaccinated (IUV, square markers) and Incidence in Vaccinated (IV, triangle markers) were estimated by SNVCS survey period, for the overall period between 2005-2022, and for the overall period between 2008-2022 (due to the change in TBE vaccination recommendations in 2006). (B) The ratio of IUV/IV by SNVCS survey period, for the overall period between 2005-2022, and for the overall period between 2008-2022. (C) The estimated number of TBE cases prevented among children 0-17 by complete (3+ doses) TBE vaccination (square markers), as well as the number of cases that could have been prevented had 100% of children aged 0-17 been completely vaccinated against TBE (triangle markers), by SNVCS survey period, for the overall period between 2005-2022, and for the overall period between 2008-2022.
